# Editorial for the Fragile X Syndrome Genetics Special Issue: May 2023

**DOI:** 10.3390/genes14061148

**Published:** 2023-05-25

**Authors:** David E. Godler, William T. Brown

**Affiliations:** 1 Diagnosis and Development, Murdoch Children’s Research Institute, Royal Children’s Hospital, Melbourne, VIC 3052, Australia; 2 Department of Paediatrics, Faculty of Medicine, Dentistry and Health Sciences, University of Melbourne, Parkville, VIC 3052, Australia; 3 Central Clinical School, Sydney Medical School, University of Sydney, Camperdown, NSW 2006, Australia; wtbibr@aol.com; 4 Institute for Basic Research in Developmental Disabilities, Staten Island, NY 10314, USA

Fragile X syndrome (FXS) is the leading single-gene cause of inherited intellectual disability and autism. A CGG trinucleotide expansion of greater than 200 repeats, known as a full mutation (FM), usually causes FXS. It is located in the promoter of the *FMR1* gene, and it usually induces epigenetic silencing and loss of the *FMR1* protein product, FMRP. FMRP is an RNA-binding protein essential for normal neurodevelopment involved in the regulation of synaptic plasticity and neuronal function. This Special Issue comprises a clinical component focusing on co-morbidities of FXS including one review, two original research articles, one case report and a molecular component which includes one review, two original articles and two case reports.

For the clinical component, Cregazan-Royo et al. [1] present a systemic review of the current state of knowledge on behavior and social competence issues in FXS. This review assesses three databases leading to fifty-one studies published in the last 20 years. It identifies attention-deficit/hyperactivity disorder (ADHD) problems as the greatest behavior issue reported for FXS. The authors conclude that while the socialization trajectory from childhood to adolescence in FXS remains unclear, comorbidity with autism in FXS is associated with increased behavior problems and worsened social competence profiles. Moreover, environmental factors and parental characteristics are identified as major influences of behavior problems and social competence. 

Lozano et al. [2] present a survey of the presence, frequency and duration of anxiety-related symptoms in a large cohort of individuals affected with FXS from 2 to 81 years of age. The authors conclude that while anxiety is a challenging endpoint to report as an outcome measure for individuals with FXS, caregivers are capable of observing and reporting behaviors that are valid indicators of anxious states. The study utilizes standardized self-report-based assessments, with the results supporting the development of an anxiety measure for FXS for the benefit of caregivers. This would address issues associated with rater inference. Shuleski et al. [3] explores the concerns and challenges faced by caregivers of individuals affected with FXS. The study administers an anonymous online survey to find greater than 70% of caregivers with concerns about the future of individuals with FXS whom they cared for, with most concerns regarding the future need for support of younger individuals. Focusing on the physiological issues of FXS, Lewis et al. [4] present two adult males with FXS displaying the aortic aneurism characteristic of Marfan syndrome. The authors conclude that while it is not uncommon for individuals with FXS to be affected with connective tissue disorders including mitral valve prolapse, this is the first report of aortic aneurisms in FXS. Aortic pathologies such as aortic aneurisms require immediate medical attention and are potentially life threatening. For these reasons, the authors suggest that monitoring of aortic aneurisms should be performed for patients with FXS with significant aortic dilatation.

For the molecular component, Hayward and Usdin [5] review the current state of knowledge on the mechanisms of somatic and germline instability of expanded *FMR1* alleles in Fragile X-related disorders. The review discusses the potential impact of mosaicism for *FMR1* alleles of different CGG expansion sizes, on clinical presentation. They suggest that this depends on the proportions of these expanded alleles present in affected tissues. They discuss the implications of the mechanisms related to CGG expansion instability within the *FMR1* promoter to other genes with CGG expansions and related disorders. Continuing with the theme of mosaicism, Gómez Rodríguez et al. [6] describe the clinical manifestations of an unusual case of an adult male with FXS showing an entire hemizygous deletion of the *FMR1* gene caused by maternal mosaicism. The authors place their finding in the context of the broader literature including other studies reporting mosaicism for deletions within *FMR1* and the implications of these findings for genetic counselling of women undergoing prenatal testing. They conclude that tailored profiling is necessary for accurate risk assessment in families with clinically defined de novo mutations as part of parental testing for FXS mutations. Pandelacher et al. [7] also discusses implications for prenatal testing of somatic mosaicism. The study describes a female with an *FMR1* premutation (PM) expansion and family history of FXS, referred for prenatal testing. This female is at high risk of having a child with an FM affected with FXS. This study identifies only mosaic PM alleles in this female and her male cultured chorionic villus sample (CVS) using PCR-based commercial kits, which are typically utilized in diagnostic testing. However, Southern blot analysis shows mosaicism for PM and FM alleles in both this female and her CVS. Based on these results, the authors conclude that Southern blot-based analyses should be included in pre- and postnatal testing for the presence of an FM, which may go undetected in such mosaic cases using PCR-based commercial kits. The authors also perform follow-up testing using *FMR1* methylation analyses and cytogenetic karyotyping in this female, which identifies mosaicism for 45,X0/46,XX/47,XXX lines (mosaic Turner syndrome) in the female referred for prenatal testing. 

On the theme of co-occurring diagnoses, Tabolacci et al. [8] describe molecular and clinical features of three independent rare cases with dual diagnosis of FXS and other genetic conditions including Duchenne muscular dystrophy (DMD), PPP2R5D-related neurodevelopmental disorder and 2p25.3 deletion. These patients have clinical features which appear to be modified by FXS and vice versa by another genetic condition diagnosed. The authors conclude that unusual and rare cases with clinical features not entirely consistent within the typical FXS phenotype should prompt further investigations for co-occurring conditions, rather than stopping at the first genetic diagnosis from genomic testing. Tekendo-Ngongang et al. [9] also discuss diagnostic implications and describe associations with pathogenocity for rare coding and noncoding variants in *FMR1* identified through genomic testing. The authors describe *FMR1* deletions that occur in both patients mosaic for FM alleles and as constitutional pathogenic alleles. These findings suggest that the CGG repeat region may be prone to genomic instability even in the absence of the CGG repeat expansion. The study concludes that diagnostic testing for *FMR1*-related indications such as intellectual disability should not stop at CGG sizing, but it should include methods for the detection of small coding, noncoding and copy number variants within *FMR1*.

Defining the functions of FMRP in human tissues has limitations, especially in preclinical studies. Adaev et al. [10] develop an FMRP assay to be used in mouse models of FXS to address these limitations. The study characterized an array of FMRP-specific antibodies for quantitative assessments in mouse tissues using an Enzyme-Linked Immunosorbent Assay (ELISA). The authors report on the changes in expression of FMRP using the ELISA assay between different regions of the mouse brain, different sexes and over time at four time points selected to reflect developmental differences. They conclude that the ELISA method developed and applied in this study may provide a cost-effective avenue for high-throughput analysis and direct monitoring of changes in FMRP in mouse tissues. Importantly, this assay has potential applications in gene therapy efficacy or treatment surveillance trials in mouse models of FXS, which may be required prior to human efficacy trials. 

In line with this topic, Chadman et al. [11] report on the feasibility of a gene therapy strategy in a mouse model of FXS. The study uses an Adeno-associated virus (AAVs) strategy, which has previously been extensively employed for gene transfer to the nervous system. The study tests if peripheral administration of the *FMR1* construct using this strategy to adult wild-type and *FMR1* knock-out (KO) mice would lead to central expression of *FMR1* and improvements in the behaviors associated with FXS in the KO model. The authors report that the gene delivery is highly efficient using this system, and that it exceeds the control FMRP levels in all tested brain regions. The authors also observe improvements of FXS-related phenotypes in the KO model as a result of administration including performance in the rotarod test. Based on these results, the authors conclude that the strategy demonstrated efficient, brain-specific delivery of *FMR1* via peripheral administration in adult mice, with improvements in a number of FXS-related phenotypes. However, the authors also caution regarding the use of these vectors in humans, where they are less efficient than in mice. They suggest that studies to determine the optimal dose using human-suitable vectors will be necessary to further demonstrate feasibility.

The articles included in this Special Issue provide novel clinical and molecular insights into the genetics and genomics of FXS with direct implications for improved screening, diagnosis and treatments for affected individuals. Together, these works highlight gaps in our current knowledge and their implications for current clinical and diagnostic practice. They focus on somatic mosaicism, dual diagnoses, deletions and sequence variations to *FMR1* identified by genomic testing that may not be identified by standard-of-care testing for FXS. The development of high-throughput analysis of FMRP in mouse tissues and testing of efficacy and feasibility of a gene therapy approach in a mouse model of FXS complete this Special Issue. We anticipate that these contributions will lead to an improved understanding of the neuro-pathobiology and developmental trajectories of the disease, which may ultimately lead to the development of new therapeutics. 
